# Primary Isolated Extramedullary Plasmacytoma in the Colon

**DOI:** 10.4021/gr552w

**Published:** 2013-09-09

**Authors:** Seung-Hyun Lee, Byung-Kwon Ahn, Sung-Uhn Baek, Hee-Kyung Chang

**Affiliations:** a Department of Surgery, Kosin University College of Medicine, Busan, Korea; b Department of Pathology, Kosin University College of Medicine, Busan, Korea

**Keywords:** Colon, Plasmacytoma, Treatment

## Abstract

Primary isolated extramedullary plasmacytoma is a rare tumor. Although it commonly involves nasopharynx or upper respiratory tract, only 10% of cases involves the gastrointestinal tract. Stomach and small intestine are the most commonly involved sites in the gastrointestinal tract. Primary isolated extramedullary plasmacytoma of colon is extremely rare. We report a case of 45-year-old man who presented with 1-year history of lower abdominal pain. Colonoscopy showed a colonic stricture about 50 cm from the anal verge. Colonoscopic biopsy showed lymphoid hyperplasia. On computed tomography, enhancing circumferential wall thickening and luminal narrowing with pericolic lymph node enlargement in the transverse colon was identified. Patient underwent extended left hemicolectomy. Histopathologic examination of resected colon identified an isolated primary colonic plasmacytoma of 1.7 cm in diameter with regional lymph node involvement (8/50 positive). To administer adequate treatment, further study about clinical features of primary isolated extramedullary plasmacytoma of colon is necessary.

## Introduction

Plasma cell tumor is an immunoproliferative monoclonal disease of the B-cell line that originates from malignant transformed plasma cells. It is composed almost exclusively of plasma cells that are arranged in clusters or sheets with a scant, delicate, supportive, connective tissue stroma. Plasmacytoma and multiple myleloma are two main groups in plasma cell tumors. Plasmacytoma includes solitary plasmacytoma of bone and solitary extramedullary plasmacytoma.

Solitary extramedullary plasmacytoma has been reported rarely and are not very well defined about natural history and diagnosis. Mostly it has been found in nasopharynx or upper respiratory tract, only 10% of cases involves the gastrointestinal tract. Stomach and small intestine are the most commonly involved sites in the gastrointestinal tract [1-3]. Primary isolated extramedullary plasmacytoma of the colon is extremely rare. Here we report a rare case of a primary isolated extramedullary plasmacytoma of the colon and its clinical course after surgery.

## Case Report

A 45-year-old man was admitted with 1-year history of intermittent abdominal pain on lower and periumbilical abdominal portion. Abdominal pain had been mild, dull nature, but was aggravated 3 months ago. It was associated with occasional vomit and diarrhea. There was history of weight loss of 2 kg in last 3 months. There was no history of fever, anorexia, or alternating constipation. Past medical history was not remarkable.

The patient was noted to have a blood pressure of 130/80 mmHg, a pulse rate of 70 beats/min, and body temperature of 36.5 °C. Physical examination revealed mild tenderness without muscle rigidity on periumbilical abdominal portion, but no palpable abdominal or rectal mass. None of the lymph nodes were palpable from the body surface.

Laboratory examination on admission reveals that white blood count was 6,100/µL, hemoglobin was 14.7 g/dL, hematocrit was 42.8%, and platelet was 314,000/µL. Serum carcinoembryonic antigen and CA 19-9 were within their normal ranges. An abdominal X-ray showed ileus pattern with focal small bowel distension. Colonoscopy showed a colonic luminal protruding mass-like lesion about 50 cm from the anal verge. From the anal verge 45 - 55 cm, edematous and nodular mucosal change was identified. Colonoscopic biopsy showed lymphoid hyperplasia. On comuted tomography, enhancing circumferential wall thickening and luminal narrowing with pericolic lymph node enlargement in the transverse colon was identified.

At laparoscopic laparotomy, a mass lesion involving the descending colon was identified. There was no evidence of intra-abdominal tumor spread. A laparoscopic extended left hemicolectomy with lymph node dissection was performed.

**Figure 1:**
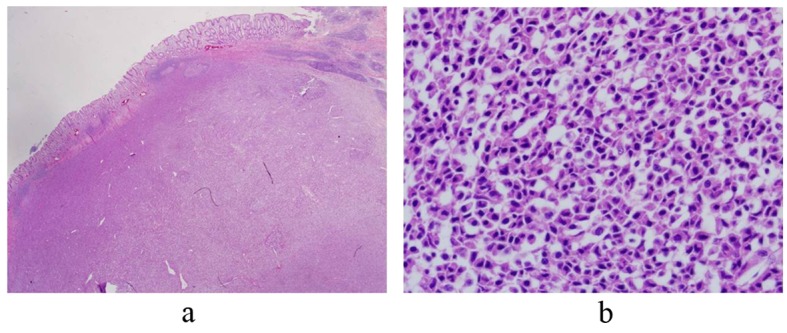
The colon wall is effaced by discrete plasma cell tumor. (H&E, × 40) (a) The tumor cells show abundant cytoplasm and eccentric nuclei with coarse chromatin (b).

**Figure 2:**
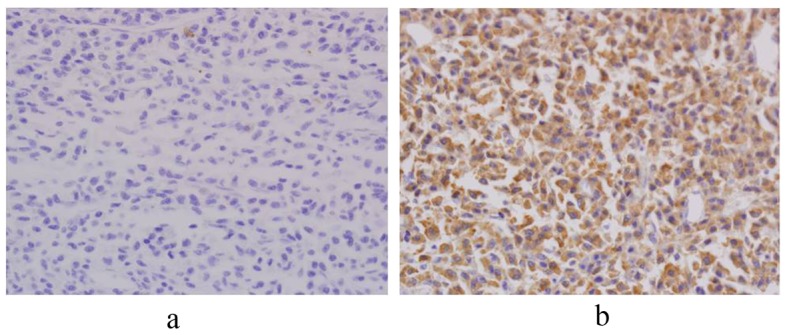
Immunohistochemistry shows monoclonality for lambda light chain. The expression of lambda light chain is seen. However, it of kappa light chain is not seen. (Immunohistochemistry, × 200).

On gross examination of specimen, an ill-defined tumor (6.5 × 4.5 cm) with near circumferental serosal nodularity was identified. The cut surface of the tumor was flesh like, pinkish and gray colored. It showed focal localized oval mass (1.7 × 1.5 cm) in mucosa. Histopathologic examination showed that the oval mass was composed of a diffuse proliferation of plasma cells, and invaded muscle propria layer of the colon wall. Lymphoid proliferation is dispersed throughout the uninvolved area as well as around the mass (Fig. 1a, b). The surgical margins were free from tumor cells. Eight lymph nodes were positive for tumor cells (8/50 positive). Immunohistochemistry study showed that lambda right chain was positive (Fig. 2a, b). Other markers (CK, CK20, Vimentin, LCA, CD20, CD3, CD45RO, kappa right chain) were negative. Based on pathological examination, a diagnosis of plasmacytoma of colon was made. To exclude associated multiple myeloma, patient underwent bone marrow biopsy, bone imaging survey. His peripheral blood smear, serum protein electrophoresis, and urin immunoelectrophoresis for Bence-Jones protein were found to be normal.

Postoperatively, patient discharged without any complications. Patient did not undergo postoperative adjuvant chemotherapy and has been on regular follow up in last 36 months without recurrence.

## Discussion

Extramedullary plasmacytoma accounts for only 3-5% of all plasma cell disease. They may be solitary or may precede, accompany or follow the onset of multiple myeloma. Solitary extramedullary plasmacytoma has been reported rarely and are not very well defined about natural history and diagnosis. Diagnosis of solitary extramedullary plasmacytoma requires the exclusion of associated multiple myeloma as shown by negative Bence-Jones protein in urine, normal serum electrophoresis and normal bone marrow biopsy [4]. Our present case met the above criteria.

Most of extramedullary plasmacytoma has been found in nasopharynx or upper respiratory tract, only 10% of cases involves the gastrointestinal tract. Stomach and small intestine are the most commonly involved sites in the gastrointestinal tract [1-3]. Primary isolated extramedullary plasmacytoma of the colon is extremely rare [5-13]. So, its clinical features and prognosis were not well known. There is currently no general guideline for treatment of patients with primary isolated extramedullary plamacytoma of the colon.

Primary isolated extramedullary plasmacytoma of the colon affects more commonly men than female (3:1). Patients in the age group of 35 to 85 years are usually affected (mean, 50.9, range 15 - 90). This age group is somewhat younger than those for multiple myeloma or colon carcinoma, which have mean ages of 60 - 70 years. In the colon and rectum, the most common involving site was the cecum.

Clinical presentation is variable with abdominal pain, intestinal bleeding and diarrhea. Duration of symptoms varied from a few weeks to as long as several years. In the present case, patient was 45 years old, and presented 1-year standing abdominal pain with occasional diarrhea. Clinical presentations may mimic that of carcinoma, intestinal tuberculosis or inflammatory bowel disease. Differential diagnosis is important for management. Bak et al [14] reported a case with extramedullary plasmacytom in the sigmoid colon, which underwent total colectomy with a preoperative diagnosis of ulcerative colitis.

Barium enema examination commonly showed polypoid mass or constricting lesion with or without mucosal or submucoal infiltration [10]. Colonoscopy showed polyps, a non passable stricture, but biopsy taken from stricture site did not reveal any special pathologic findings [5, 9, 10]. These colonic lesions may mimic adenocarcinoma on radiologic findings. In present case, preoperative diagnosis could not get properly. Colonoscopic biopsy showed lymphoid hyperplasia. Surgery was performed under a diagnosis of carcinoma or lymphoma.

Although plasma cell tumors are known to be radiosensitive, surgical treatment is undoubtedly the most frequent treatment choice for the primary isolated extramedullary plasmacytoma in the colon. In some cases involving the rectum, radiotherapy was administered for local control or palliative intention [15]. Chemotherapy has no effect on the course of extramedullary plasmacytoma. However, it is the treatment of choice for disseminated disease and can be also used preoperatively to shrink tumors.

Prognosis and long term follow up of primary isolated extramedullary plasmacytoma in the colon were not well known. Gupta et al [10] reported no recurrent case at 17-months follow up, which had tumor invading all layer of the colon with 3 lymph node metastases, underwent postoperative adjuvant chemotherapy. However, Doki et al [11] reported a recurrent case, 4 month after operation. Chemotherapy was not effective for that case. The patient died just 6 months after the recurrence. Prognosis of extramedullary plasmacytom is generally good. Liebross et al [1] reported that the median survival was 9.5 years and 56% patients were free from systemic disease at 5 years. One third of extramedullary plasmacytoma had multiple myeloma after a median follow up of 1.8 years. In this present case, there was no recurrence and conversion to multiple myeloma.

As primary isolated extramedullary plasmacytoma in the colon is very rare, clinical course, treatment guideline, prognosis is not very well defined. To administer adequate treatment, further study about clinical features of primary isolated extramedullary plasmacytoma of colon is necessary.
